# Wing length and host location in tsetse (*Glossina* spp.): implications for control using stationary baits

**DOI:** 10.1186/s13071-018-3274-x

**Published:** 2019-01-11

**Authors:** John Hargrove, Sinead English, Stephen J. Torr, Jennifer Lord, Lee Rafuse Haines, Cari van Schalkwyk, James Patterson, Glyn Vale

**Affiliations:** 1 SACEMA, University of Stellenbosch, Stellenbosch, South Africa; 2 School of Biological Sciences, University of Bristol, Bristol, UK; 3 Liverpool School of Tropical Medicine, Liverpool, UK; 4 London School of Hygiene and Tropical Medicine, London, UK; 5 Natural Resources Institute, University of Greenwich, London, UK

**Keywords:** Tsetse *Glossina*, Eradication using targets, Stationary and mobile baits, Wing length, Age season annual effects

## Abstract

**Background:**

It has been suggested that attempts to eradicate populations of tsetse (*Glossina* spp.) using stationary targets might fail because smaller, less mobile individuals are unlikely to be killed by the targets. If true, tsetse caught in stationary traps should be larger than those from mobile baits, which require less mobility on the part of the flies.

**Results:**

Sampling tsetse in the Zambezi Valley of Zimbabwe, we found that the number of tsetse caught from stationary traps, as a percent of total numbers from traps plus a mobile vehicle, was ~5% for male *G. morsitans morsitans* (mean wing length 5.830 mm; 95% CI: 5.800–5.859 mm) and ~10% for females (6.334 mm; 95% CI: 6.329–6.338 mm); for *G. pallidipes* the figures were ~50% for males (6.830 mm; 95% CI: 6.821–6.838 mm) and ~75% for females (7.303 mm, 95% CI: 7.302–7.305 mm). As expected, flies of the smaller species (and the smaller sex) were less likely to be captured using stationary, rather than mobile sampling devices. For flies of a given sex and species the situation was more complex. Multivariable analysis showed that, for females of both species, wing lengths changed with ovarian age and the month, year and method of capture. For *G. pallidipes*, there were statistically significant interactions between ovarian age and capture month, year and method. For *G. m. morsitans*, there was only a significant interaction between ovarian age and capture month. The effect of capture method was, however, small in absolute terms: for *G. pallidipes* and *G. m. morsitans* flies caught on the mobile vehicle had wings only 0.24 and 0.48% shorter, respectively, than flies caught in stationary traps. In summary, wing length in field samples of tsetse varies with ovarian age, capture month and year and, weakly, with capture method. Suggestions that a target-based operation against *G. f. fuscipes* in Kenya caused a shift towards a smaller, less mobile population of tsetse, unavailable to the targets, failed to account for factors other than capture method.

**Conclusions:**

The results are consistent with the successful use of targets to eradicate populations of tsetse in Zimbabwe. Until further, more nuanced, studies are conducted, it is premature to conclude that targets alone could not, similarly, be used to eradicate *G. f. fuscipes* populations in Kenya.

**Electronic supplementary material:**

The online version of this article (10.1186/s13071-018-3274-x) contains supplementary material, which is available to authorized users.

## Background

The host-orientated behaviour of tsetse (*Glossina* spp.) is important in determining the epidemiology of the potentially fatal diseases caused by the blood parasites (*Trypanosoma* spp.) which male and female tsetse can transmit when feeding on humans and livestock [1–4]. Vale et al. [5] suggested that the distinctive behaviour patterns of different tsetse species, and of the two sexes, are markedly affected by the mobility of the flies, which is increased when the habitat is extensive and flies are large. The sorts of behaviour affected include: (i) the relative probability of being caught by stationary as against mobile baits; (ii) the reliance on odours rather than visual stimuli in host detection; (iii) the probability of alighting on vertebrate hosts, or artificial baits; and (iv) the species of host selected. Each of these behavioural effects is subject to various, sometimes competing, considerations.

On one hand, if a fly stays still, it can find only mobile hosts that pass by, whereas the more it moves the greater the chance that it will discover more hosts of any sort, but especially more stationary hosts. The greater the number of hosts discovered, the more the flies can afford to be selective in the type of host on which they attempt to feed. Close-range responses by the fly can also be geared to making the appropriate selection, as against alighting and probing at the first opportunity in high risk situations, for example if there are bembicid wasps or asilid (robber) flies in attendance, or if the potential host itself is likely to kill the fly, as in the case of humans and baboons.

On the other hand, active searching increases the rate at which energy is utilised by perhaps 100-fold [6]. It is reasonable to suppose that considerations of energy conservation might be more serious for smaller species of flies, in accord with the findings of Vale et al. [5]. The two host-locating options are not mutually exclusive: Brady’s laboratory experiments [7, 8] showed that both spontaneous activity and responses to a mobile object increase in intensity with time since last feeding. Tsetse may feed off mobile baits early in the hunger cycle, if the feeding risk is low and little flight activity is required [9, 10]. Later in the hunger cycle, having failed to feed off a passing host, flies show higher levels of spontaneous activity and have a relatively higher probability of locating hosts.

Vale et al. [5] did not consider whether, within a given sex and species, host-orientated behaviour patterns might also be a function of fly size. Such an effect has, however, been suggested for *Glossina fuscipes fuscipes* Newstead in Kenya [11]. The core evidence was that, prior to a 19-month control campaign using stationary insecticide-treated targets on Big Chamaunga Island [12], the wing sizes of sampled females were 1% larger than they were four years later.

It was suggested that: (i) targets selectively killed larger flies within the initial population; (ii) this selection in favour of genetically and phenotypically smaller, less mobile, flies might have left a remnant population that could not be eradicated using targets; (iii) such an effect might explain the claimed rarity of reports of the successful elimination of tsetse populations using targets alone [13, 14]; and (iv) the change in fly size could alter the epidemiology of tsetse-borne diseases by modifying the patterns of feeding.

The above suggestions lead to the testable hypothesis that tsetse of a given sex and species captured at a stationary bait should be larger, on average, than those captured at a mobile bait, since the latter requires a lesser demand on the flies’ flight capability. The hypothesis would become more convincing if the size variation due to bait type were great in relation to the variations associated with different taxa and with non-genetic background factors that are not related to control campaigns. To test this hypothesis, we studied the numbers and mean wing lengths of male and female *G. m. morsitans* Westwood and *G. pallidipes* Austen, sampled at Rekomitjie Research Station, Zimbabwe, using stationary odour-baited traps and a mobile vehicle-mounted electric target. We first investigated whether, indeed, the relative probability of capture by mobile and stationary baits differed between the sexes and species, according to mean wing length. Next, we assessed whether the capture probability also differed between the larger and smaller individuals within a single taxon. Finally, we explored the background variations in size due to season, year and fly age and interactions between these factors.

## Methods

The study was carried out at Rekomitjie Research Station, Zambezi Valley, Zimbabwe (16°10'S, 29°25'E, altitude approximately 520 m). The station is located in the Mana Pools National Park, which, together with the Sapi, Hurungwe and Chewore Safari Areas, has a total area of nearly 10,000 km^2^. Since 1958 there has been no agricultural settlement in this area, which was designated a UNESCO World Heritage Site in 1984, and is protected against settlement, agriculture, illegal hunting and logging. There has been no form of tsetse control in the area, nor has the area been subject to other deliberate environmental or sociological change since it was declared a National Park. Analyses of vegetation cover [15] and of game numbers [16] suggest that the Rekomitjie area continues to provide highly suitable habitat for tsetse.

The data used in this study form a subset of the results of an 11-year sampling study carried out between September 1988 and December 1999 [15]. The subset consists of flies captured in the 6-year period from January 1989 to December 1994, chosen because these were the only years when flies were captured using both stationary and mobile sampling devices. The particular devices used for the main part of the study were: (i) stationary mechanical ‘epsilon’ traps [17] baited with artificial host-odour consisting of acetone, 1-octen-3-ol, 3-*n*-propyl phenol and 4-methyl phenol released at ~ 200, 0.4, 0.01 and 0.8 mg/h, respectively; and (ii) a vehicle-mounted electric target (VET), which consisted of an electrocuting grid, 1m tall and 2m long [18] mounted on the back of an open pickup truck [19]. For brevity these two systems are referred to below as “trap” and “VET”, respectively. For further details of these sampling devices, and of artificial refuges used in the collection of ancillary data, see Additional file 1: Figures S1-S3 and associated text in Additional file 1: Text S1.

Daily maximum and minimum temperatures were recorded from mercury thermometers housed in a Stevenson screen at the Research Station. Daily rainfall was recorded using a gauge placed 4 m from the Stevenson screen.

Details of the tsetse life-cycle are provided in Additional file 1: Text S2. The total numbers of each sex and species of tsetse caught by each bait were recorded, but wing length was measured in only a random sample of captured flies. Captured flies, after transfer to the laboratory, were placed in individual (75 × 25) mm plastic tubes and placed under a black cloth to reduce activity. Females were then subjected to ovarian dissection, using the technique developed for tsetse by Saunders [20, 21] and improved by Challier [22, 23]. The procedure is described in detail by Hargrove [24] and in Additional file 1: Figure S4 and associated text in Additional file 1: Text S3. Technicians dissected flies in the order that they were provided. The ovarian dissection technique is used to gauge the number of times a female fly has ovulated, and thus provides a measure of her age. For studies of wing length, as indices of body size, wings of sampled flies were affixed with transparent sticky tape to the dissection record form and a measure of wing length (as explained in further detail below) was taken using a binocular microscope fitted with a graduated reticule in the eyepiece. Sub-samples of 979 female *G. pallidipes* caught in artificial refuges in October 1992 [25, 26] and 1752 in odour-baited traps in February 1994 [27] were subjected both to ovarian dissection and, thereafter, to nutritional analysis, to ascertain levels of fat and of the residual (i.e. fat-free) dry weights of the thorax and abdomen [26, 27]. Tsetse enter refuges only when temperatures exceed 32 °C, generally in September through November. The construction and use of the artificial refuges are fully described elsewhere [25, 26]. Climate profiles for Rekomitjie Research Station are discussed in Additional file 1: Text S4 and monthly mean maximum and minimum temperatures and rainfall are illustrated in Additional file 1: Figure S5.

**Fig. 1:**
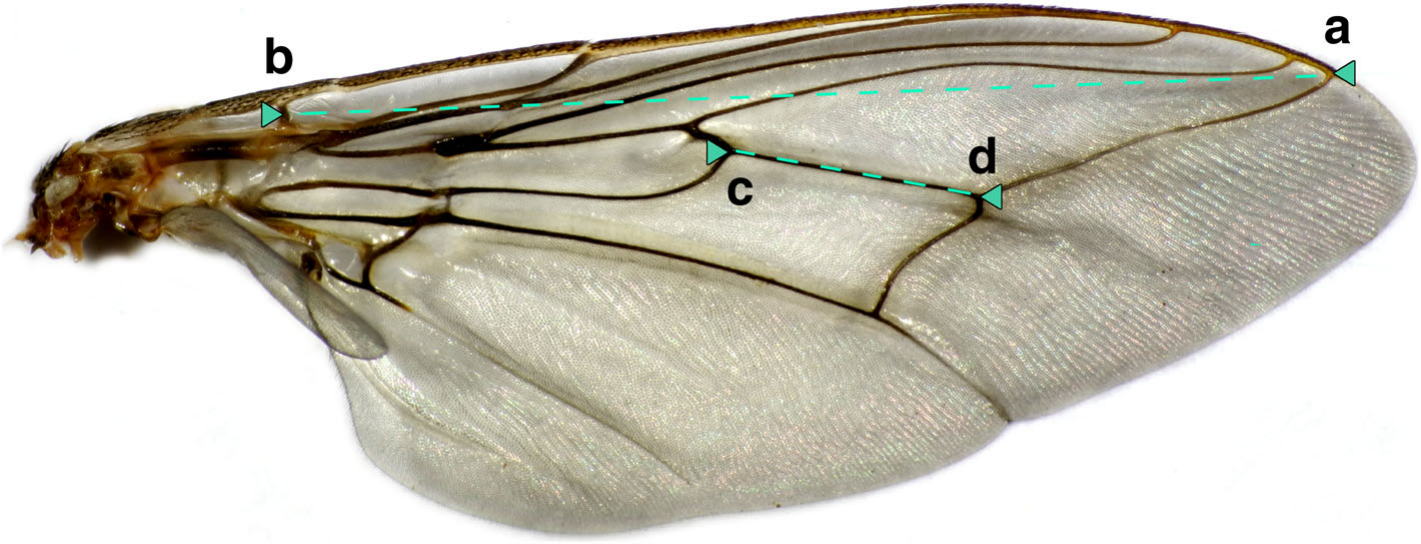
Photograph of a tsetse wing. The right wing of a female *G. m. morsitans* showing the endpoints **a** and **b** used as a measure of wing length in present studies. The hatchet cell length used in classical studies is measured between points **c** and **d**

C. H. N. Jackson instituted the practice of measuring the length of the middle part of the fourth longitudinal vein (length CD, Fig. 1), corresponding to the “cutting blade” of the hatchet cell and this practice has been followed by most tsetse workers in the past [28]. We used, instead, the distance between markers A and B (Fig. 1) because this is a longer distance, so that the relative error, arising as a result of variation about the choice of endpoints due to vein thickness, should be reduced. Measurements, all made on a single wing of each fly, were calibrated using a stage micrometer and all lengths were converted to mm.

It should be noted that Mbewe et al. [11] used, as a measure of fly size, the “centroid size” (CS) defined by Dujardin & Slice [29] as the square root of the sum of the squared distances to the centroid of each of a number of pre-defined landmarks on a wing. We have data for these landmarks for a subset of 40 wings collected in January 1994 and we compared the CS values obtained for these data with our measure of the wing lengths. We also investigated the correlation between our measure of wing length and the thoracic residual (fat free) dry weight, which has been used previously as a measure of size in tsetse [30]. These correlations are demonstrated using sub-samples of female *G. pallidipes*, captured from artificial refuges and odour-baited traps, and subjected both to ovarian dissection and, thereafter, to nutritional analysis (see above).

Data were analysed using simple and multivariable linear regression, with error limits indicated by 95% confidence intervals. All data analyses were carried out using Microsoft Excel and Statacorp Stata v.14.2.

## Results

### Measures of fly size

#### Wing length *vs* centroid size (CS)

**Fig. 2:**
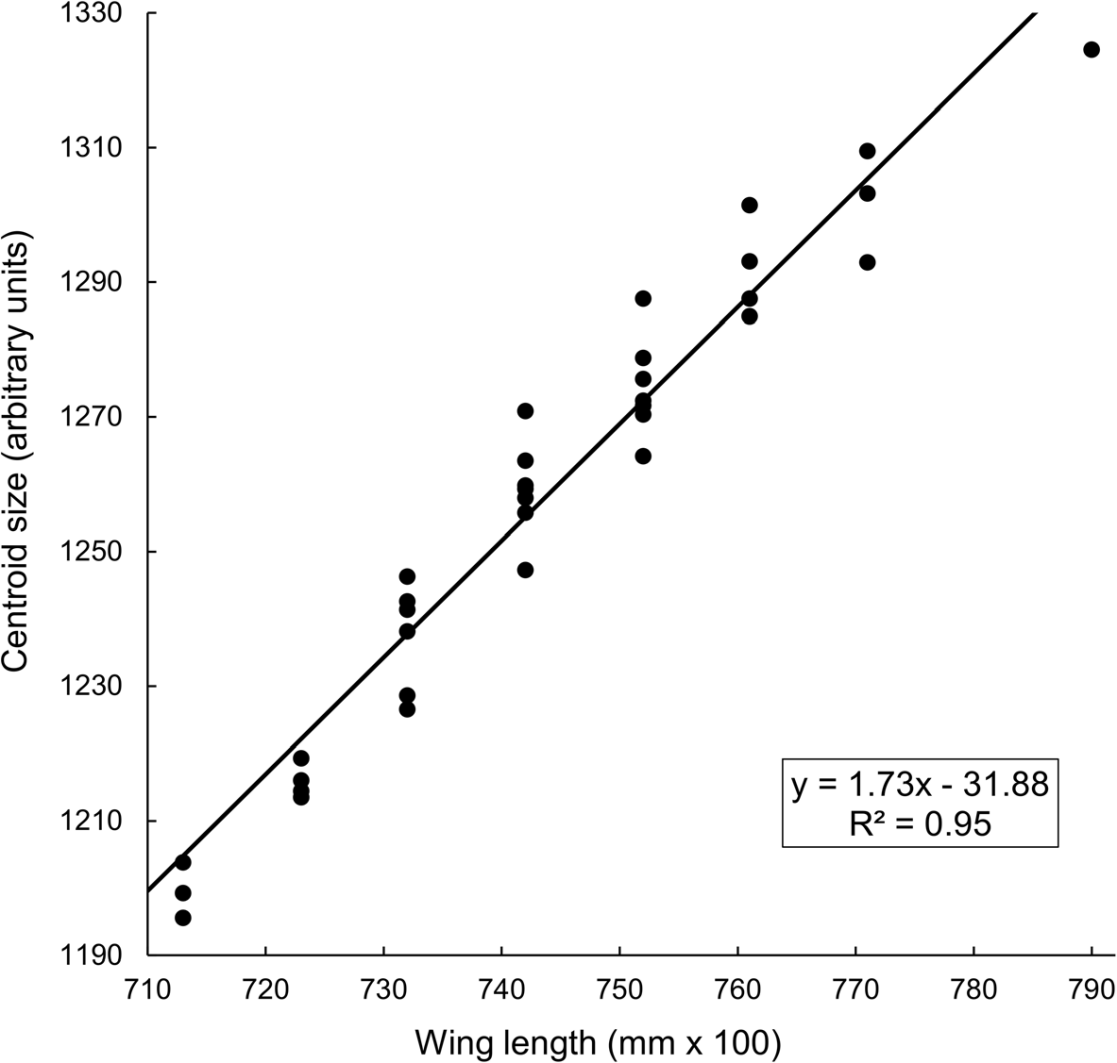
Tsetse wing lengths and centroid sizes. Centroid sizes plotted against wing lengths for 40 female *G. pallidipes* female flies sampled in January 1992. Fit by linear regression (Student’s t-test, *n* = 40, *df* = 38, *P* < 0.001)

For the wings of 40 female *G. pallidipes* captured during January 1992, the mean monthly values of CS and wing length factor were highly correlated (Fig. 2).

#### Wing length *vs* thoracic residual dry weight

Regression analysis showed that thoracic residual dry weight (TRDW) was significantly correlated with our measure of wing length for female *G. pallidipes* captured using either artificial refuges or traps (Table 1). To ensure that all flies had completed the development of their thoracic musculature and exoskeletal structures, the analyses were restricted to flies that had produced their first larva.

**Table 1:** Wing length and thoracic weight in tsetse. Linear regression of thoracic residual dry weight (TRDW) against wing length for female *G. pallidipes* captured in artificial refuges (*n* = 712) in October 1993 or odour-baited traps (*n* = 1352) in February 1994, at Rekomitjie

TRDW		Coefficient	SE	Student’s t statistic	*P*-value	95% CI
Wing length	Refuge	1.75	0.10	16.92	<0.001	1.55–1.96
	Trap	1.63	0.06	25.20	<0.001	1.50–1.75
Constant	Refuge	-6.00	0.76	-7.85	<0.001	-7.50– -4.50
	Trap	-4.76	0.47	-10.11	<0.001	-5.69– -3.84

*Abbreviations*: SE, standard error; CI, confidence interval

### Gross effects of species and sex on wing length

Table 2 shows the total numbers of flies sampled using traps, and the VET, during the study, and Table 3 shows the mean wing lengths among sub-samples of the captured flies of each sex and species in the pooled samples obtained from both baits in all seasons between January 1989 and December 1993. Since the principle interest of the overall study was in the age structure of the female population [31], the sample of flies examined was heavily biased in favour of females. Moreover, since the majority of flies were caught in traps - which capture *G. pallidipes* with a higher probability than *G. m. morsitan*s - the sample was dominated by females of the first species.

**Table 2:** Total catches of male and female *G. m. morsitan*s and *G. pallidipes* sampled using traps and the VET at Rekomitjie between January 1989 and December 1993. The bottom row shows the percentage of the total catch for which the wing length was measured. For example, the wing length was measured in 41.6% of the 208,589 female *G. pallidipes* captured

Method	*G. m. morsitans*		*G. pallidipes*	
	Males	Females	Males	Females
VET catches	16,091	10,143	14,284	19,773
Sex ratio		0.63		1.38
Species ratio			0.89	1.95
Trap catches	7230	10,575	95,274	188,816
Sex ratio		1.46		1.98
Species ratio			13.18	17.85
Total catches	23,321	20,718	109,558	208,589
% processed	1.0	53.9	3.6	41.6

**Table 3:** Mean wing lengths of subsets of the captured flies

Species	Sex	*n*	Percent	Wing length (mm)	95% CI	Percentiles	
						1%	99%
*G. m. morsitan*s	Males	238	1.0	5.830	5.800–5.859	5.47	6.65
	Females	11,162	53.9	6.334	6.329–6.338	5.75	6.83
*G. pallidipes*	Males	3954	3.6	6.830	6.821–6.838	6.14	7.48
	Females	86,675	41.6	7.303	7.302–7.305	6.68	7.81

*Abbreviations*: *n*, sample size on which the means were calculated; CI, confidence interval

*Note*: 1% of the *n*-values lie below/above the 1%/99% percentiles

Wings for females were, on average, 6.9% longer than for males in *G. pallidipes*, and 8.6% longer in *G. m. morsitan*s (Student’s t-test, *P* < 0.001 in each case). The larger difference for the latter species means that there was little overlap in the distribution of wing lengths between male and female *G. m. morsitan*s whereas the overlap was substantial for *G. pallidipes* (Table 3, 1% and 99% percentiles). For females, wings were 15.3% longer in *G. pallidipes* than in *G. m. morsitan*s; for males the difference was 17.2%. Since we will also discuss results for *G. f. fuscipes*, we note here that this species is approximately the same size as *G. m. morsitans* [32].

### Numbers of tsetse caught using the trap and VET

#### Sex and species differences

**Fig. 3:**
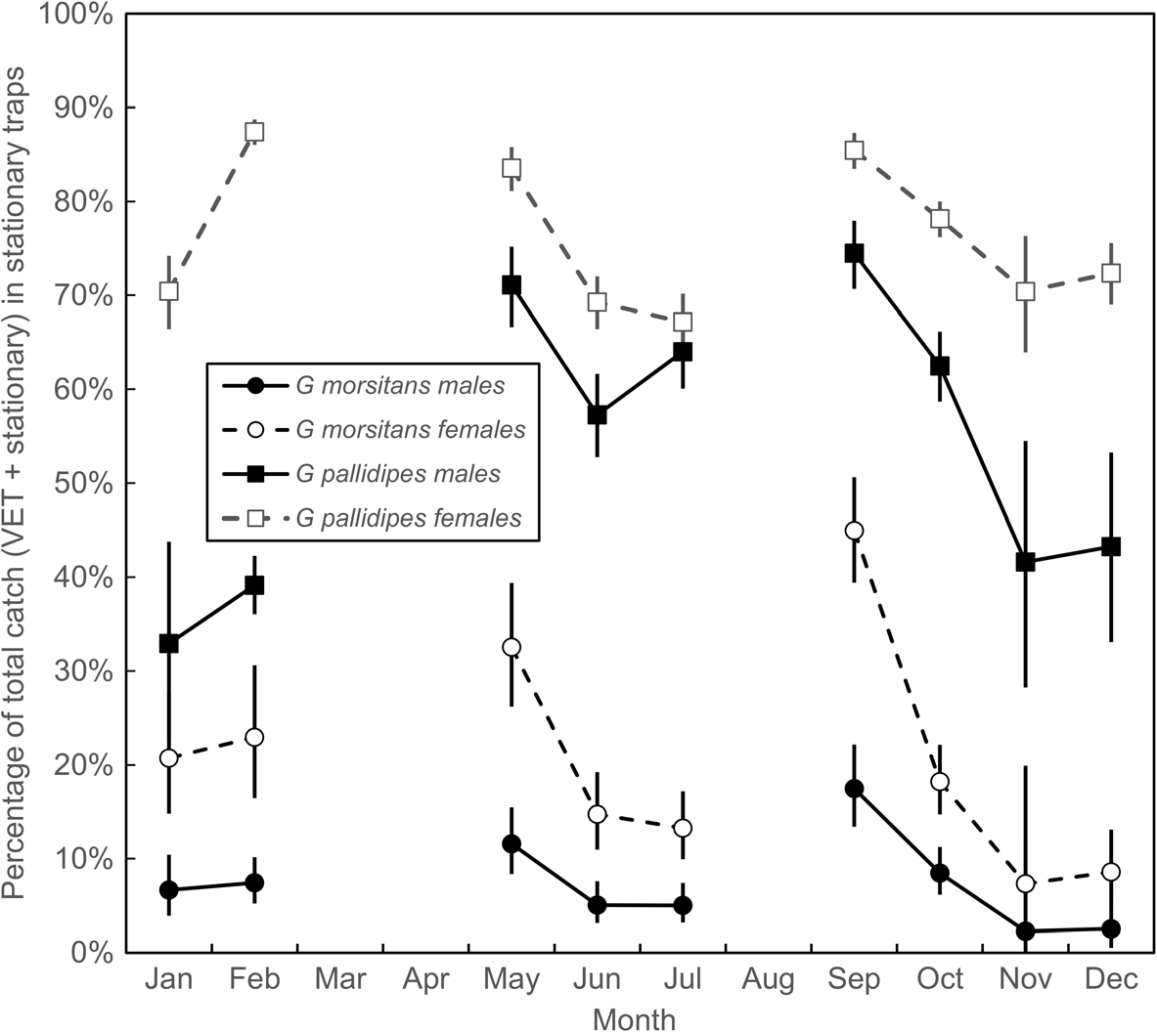
Catches of tsetse from trap and VET. Male and female *G. m. morsitan*s and *G. pallidipes* caught using stationary traps and the VET. For each month, stationary-trap catches are expressed as a percentage of the total captured by the stationary trap and the VET for that particular sex-species combination. Both devices were run in the same area at Rekomitjie during approximately the last two hours of daylight on 59 days between January and December 1992

At almost all times of the year, and among all flies caught in both sampling devices, < 10% of male *G. m. morsitan*s, the smallest flies, were captured in the stationary trap (Fig. 3). The relatively poor performance of the trap was less marked for female *G. m. morsitan*s, but the percentage of these flies caught in the trap never exceeded 50%. For *G. pallidipes* of both sexes, but particularly for females, the proportion captured in traps was greater than for *G. m. morsitan*s. Within each sex and species, the proportion captured in traps varied with month, but the relative proportions always changed in the order indicated above. For both sexes of both species the relative proportions captured in the traps decreased as temperatures increased from September through November (Fig. 3).

#### Differences between flies of different size, within a given sex and species group

**Fig. 4:**
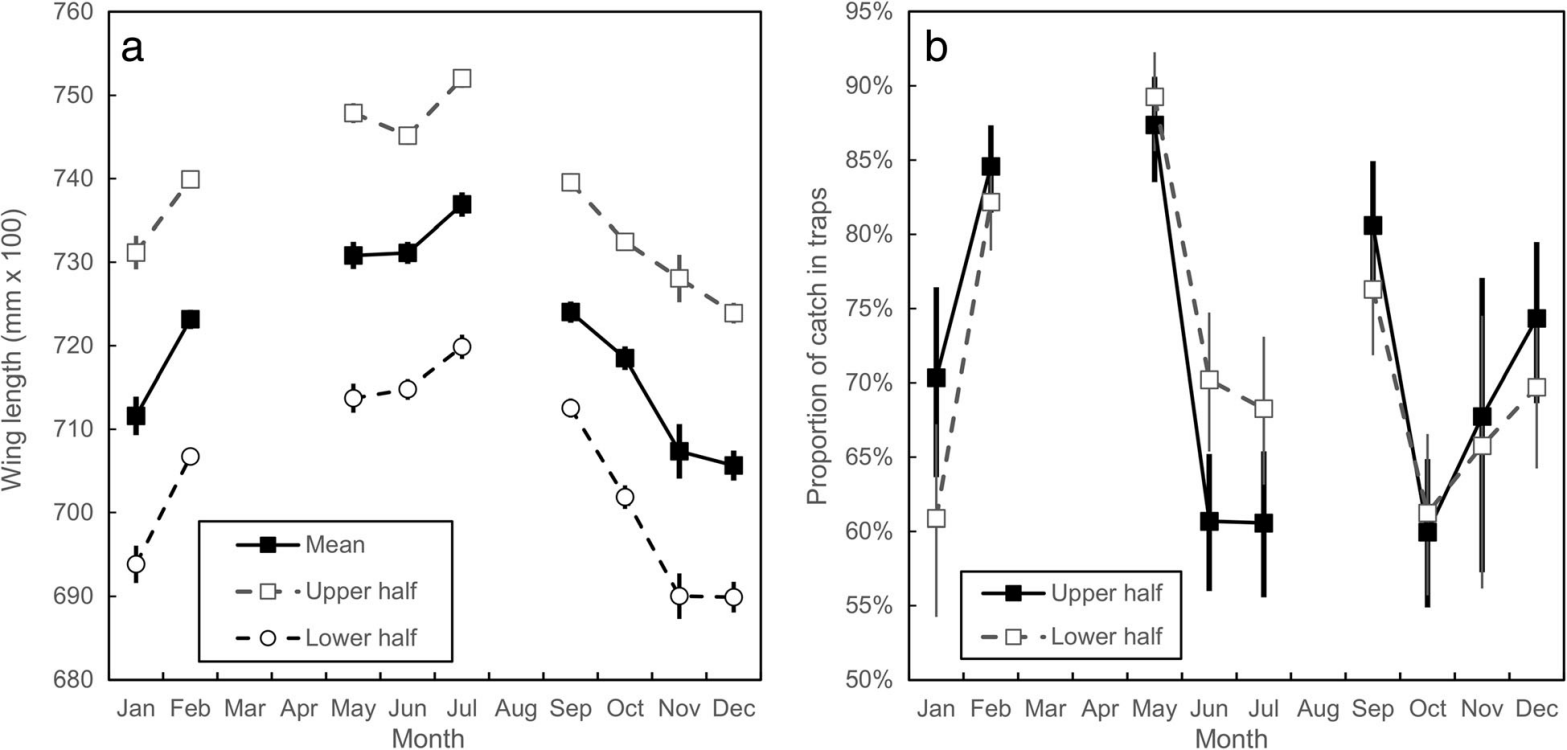
Wing lengths and numbers of female *G. pallidipes* captured using a stationary trap or VET. **a** Mean wing length for female *G. pallidipes* caught in the trap or VET at Rekomitjie, during approximately the last two hours of daylight on 59 days between January and December 1992, when both sampling systems were used on the same day in the same area. Catches were pooled by month. Means and confidence intervals are calculated for the pooled data and for the upper and lower halves of the distributions. **b** Monthly estimates for the numbers of female *G. pallidipes* caught in odour-baited traps as a proportion of the total captured using a stationary trap and the VET

The results in Fig. 3 support the hypothesis that the larger the fly, i.e. female *vs* male and *G. pallidipes vs G. m. morsitan*s, the greater is the chance the flies will be caught in a stationary trap rather than on the mobile VET. The percentage caught in traps varies with the month of the year but is generally > 10 times higher for female *G. pallidipes* than for male *G. m. morsitans*. To determine whether the same principle applies to different size ranges of flies of the same sex and species, we compared the catch distribution among all female *G. pallidipes* with wings either larger or smaller than the mean length for all flies captured on the mobile and stationary systems. Given that wing lengths fluctuate markedly with time, it was necessary in doing these comparisons to restrict the analysis to situations where the flies processed were all captured on days when the trap and the VET were both run on the same afternoon, in the same location. Figure 4a shows that the mean wing length of flies in the upper half of the distribution was 4–5% greater than those in the lower half. Figure 4b, however, shows that while, in three months, the proportion of flies caught in traps was greater for larger flies, in the other five months the reverse was true; for most months the overlap of the 95% confidence intervals showed that the proportions did not differ significantly. There is thus no reason to believe that size difference among individuals within a given sex and species group will have much impact on availability.

### Seasonal effects on wing length

**Fig. 5:**
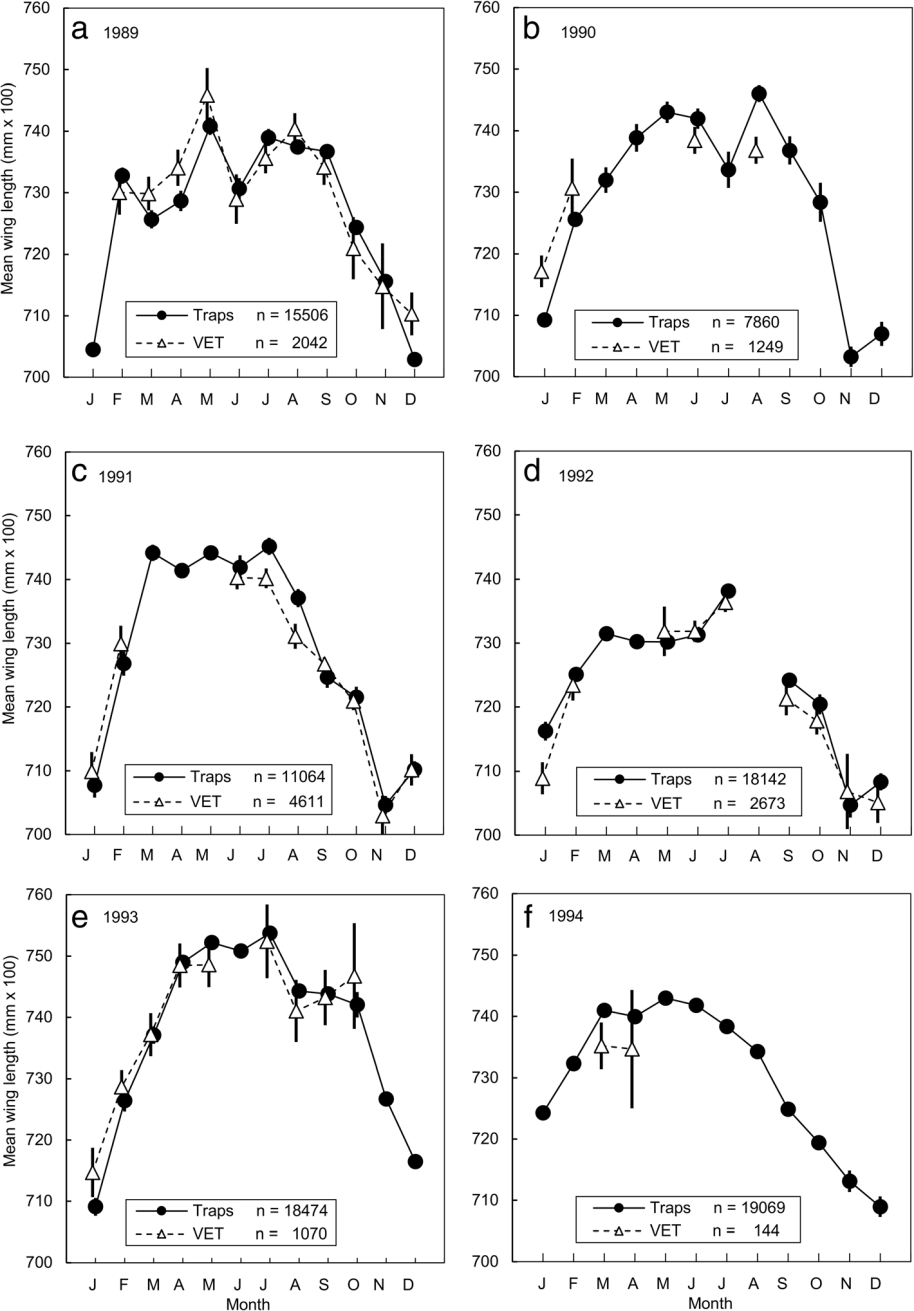
Wing length as a function of month and method of capture, for the following years: **a** 1989; **b** 1990; **c** 1991; **d** 1992; **e** 1993; **f** 1994. Monthly means of wing lengths for female *G. pallidipes* captured using odour-baited traps or a vehicle-mounted electric net (VET). Data for each capture method pooled over all sampling sites at Rekomitjie

Wing lengths for female *G. pallidipes* caught using the stationary trap and VET changed in a similar and highly predictable way with season (Fig. 5), falling steadily through the warm dry season, and increasing again once the rains started and temperatures fell. For most months, the complete overlap of 95% confidence intervals shows that there were no statistically significant differences between the wing lengths of flies captured using the two systems. Moreover, where there were significant differences, there was no consistency regarding the method that captured flies with longer wings. For example, whereas flies from the VET had significantly longer wings in March, April and December of 1989 (Fig. 5a), the reverse was the case in July and August 1991 (Fig. 5c). The pattern was the same for catches of female *G. m. morsitans*, but numbers were too low for a meaningful comparison of the month-by-month changes in catches from individual capture systems.

### Other factors affecting wing length

The preceding results suggest that mean wing lengths vary little with the way that tsetse are sampled. There are, however, at least four complicating factors. First, the age structures of samples vary significantly depending on the sampling method [31]. Secondly, the relationship between fly size and fly age also changes with season [26, 27]: in estimating the effect of capture method on wing length we should, therefore, adjust for fly age and season. Thirdly, it also appears that wing lengths change from year to year and we need to adjust for these changes. For example, the highest wing lengths in July 1992 and 1993 were 7.38 mm (95% CI: 7.37–7.39 mm) and 7.54 mm (95% CI: 7.53–7.55 mm), respectively, with no overlap of the 95% confidence intervals. Similarly, the lowest mean wing lengths in 1992, 7.05 mm (95% CI: 7.03–7.70 mm), occurred in November and were significantly shorter than the shortest lengths in 1993, 7.16 mm (95% CI: 7.15–7.18 mm) which occurred in December (Student’s t-test, *P* < 0.001 for all of the differences). Finally, there were significant interactions between some of the above-named variables.

### Wing length as a function of ovarian age: interaction with month of capture

**Fig. 6:**
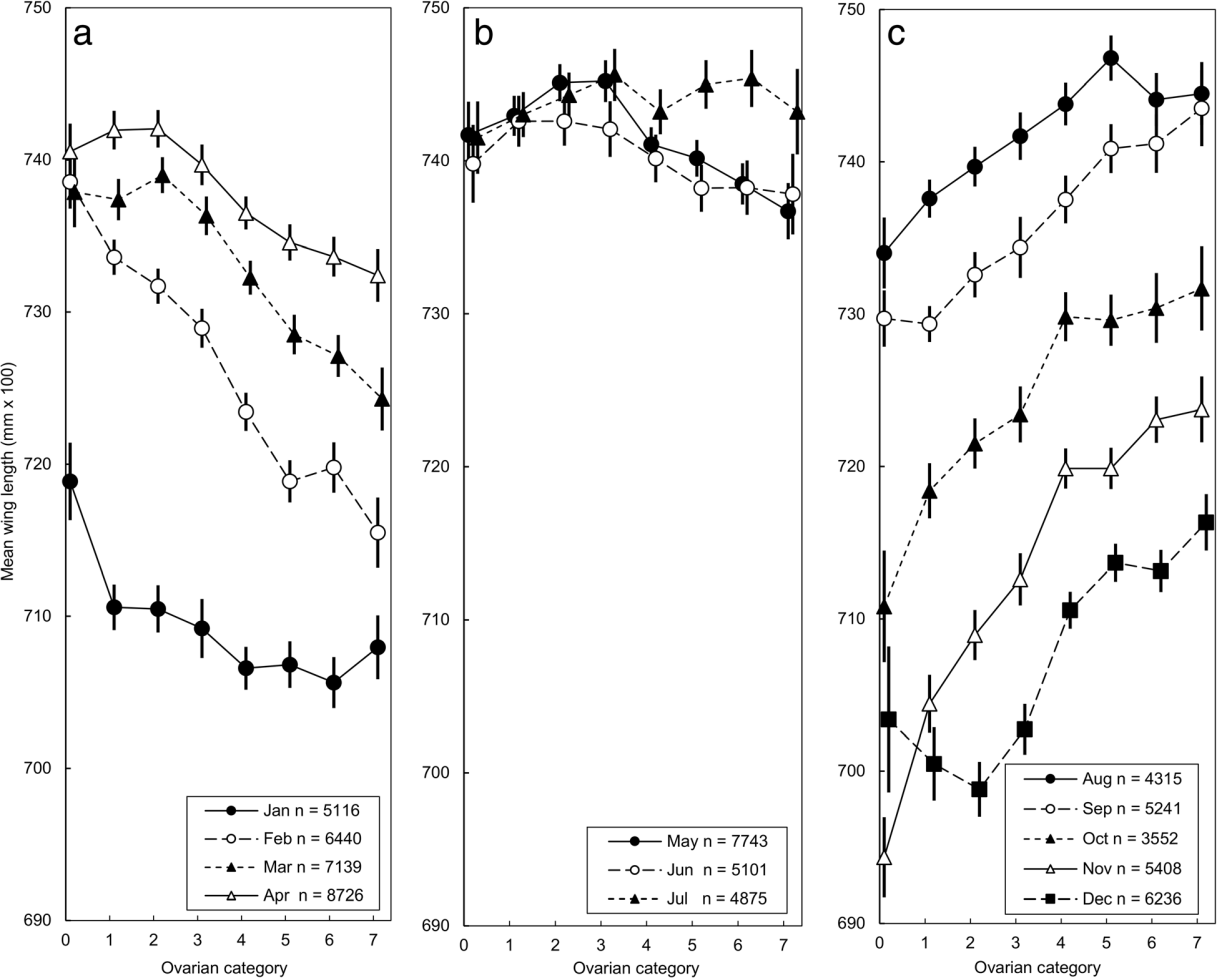
Tsetse wing lengths as a function of age and month of capture: **a** January – April; **b** May – July; **c** August – December. Mean wing lengths of female *G. pallidipes* as a function of ovarian age and month of capture. Flies caught in odour-baited traps at Rekomitjie from January 1989 to December 1993

Over the period 1959–2015, 95% of the average annual rainfall at Rekomitjie fell in the months November-March, with 73% in December-February. With the onset of the main rains in December, the size of young flies began to increase, a trend that accelerated in January and February (Fig. 6a, c). Thereafter, from March until July, there was little change in the wing lengths of flies in ovarian category 0 (Fig. 6a, b). The net result of these changes was that wing length decreased more steeply with increasing ovarian age with each successive month between December and February. This trend slowed in March-April, when there was no further increase in the size of young flies: between May and July wing length was almost independent of ovarian category (Fig. 6b). In August, when temperatures started to increase, the wing lengths of young flies began to decrease: this trend accelerated through November, with the result that wing length then increased ever more steeply with increasing ovarian age, with each successive month between August and November (Fig. 6c).

### Multivariable analysis of factors affecting wing length in females of both species

Given the foregoing results, we performed regression analyses with wing length as the dependent variable and with the following independent variables: ovarian category, capture month, capture year and capture method. As before, the data were restricted for this analysis to the period when both traps and the VET were used to sample flies (January 1989 to December 1993). Only about 5% of the data available were for males, for which there was no accurate measure of age; accordingly, we only analysed the data for female tsetse. We used likelihood ratios to test for statistically significant interactions between independent variables, comparing results of runs where interactions were, or were not, included. For *G. pallidipes*, there were statistically significant interactions between ovarian category and each of capture month, year and method. For *G. m. morsitans* there was a significant interaction between ovarian category and capture month, but not with capture year or method. The complete results of the full model analyses are shown in Additional file 1: Table S1; results for the runs where only the main effects were included are shown in Table 4.

**Table 4:** Multivariable analysis of the effects of ovarian age, month, year and method of capture on the wing length of female *G. pallidipes* (*n* = 80,219; *r*^2^ = 0.26) and *G. m. morsitans* (*n* = 7764; *r*^2^ = 0.24) captured in the field at Rekomitjie between January 1989 and December 1993

	*G. pallidipes*			*G. m. morsitans*		
	Coefficient	95% CL		Coefficient	95% CL	
		Lower	Upper		Lower	Upper
Ovarian age						
1	-1.21	-1.88	-0.54***	-2.01	-3.88	-0.13*
2	0.22	-0.44	0.88	-2.45	-4.37	-0.54*
3	0.35	-0.33	1.04	-1.26	-3.26	0.74
4	0.22	-0.43	0.87	0.94	-0.91	2.80
5	0.05	-0.61	0.72	-1.72	-3.65	0.21
6	-0.18	-0.89	0.52	-0.97	-3.09	1.14
7	-0.22	-1.03	0.60	-0.61	-3.52	2.31
Year						
90	1.67	1.10	2.25***	2.47	0.33	4.60*
91	2.27	1.79	2.76***	5.08	3.46	6.70***
92	-2.85	-3.31	-2.40***	-2.27	-4.10	-0.43*
93	11.73	11.28	12.18***	8.84	6.99	10.69***
Month						
February	18.45	17.71	19.19***	9.42	7.08	11.77***
March	24.88	24.14	25.63***	17.89	14.97	20.82***
April	27.35	26.63	28.06***	24.97	21.83	28.12***
May	30.86	30.12	31.59***	22.70	19.55	25.85***
June	29.22	28.46	29.98***	23.09	20.83	25.34***
July	32.77	32.00	33.54***	27.85	25.60	30.10***
August	27.78	26.97	28.58***	23.04	20.63	25.45***
September	22.50	21.73	23.26***	21.03	18.73	23.32***
October	15.68	14.84	16.51***	13.04	10.58	15.51***
November	3.24	2.45	4.03***	-3.27	-5.86	-0.69*
December	-1.53	-2.29	-0.78***	-5.85	-8.25	-3.49***
Method						
VET	-1.73	-2.21	-1.26***	-2.97	-4.23	-1.73***
Constant	707.8	706.9	708.6	618.3	615.6	621.1

*P < 0.05, ***P < 0.001 (Student’s t-test)

*Abbreviation*: CL, 95% confidence interval limits

**Fig. 7:**
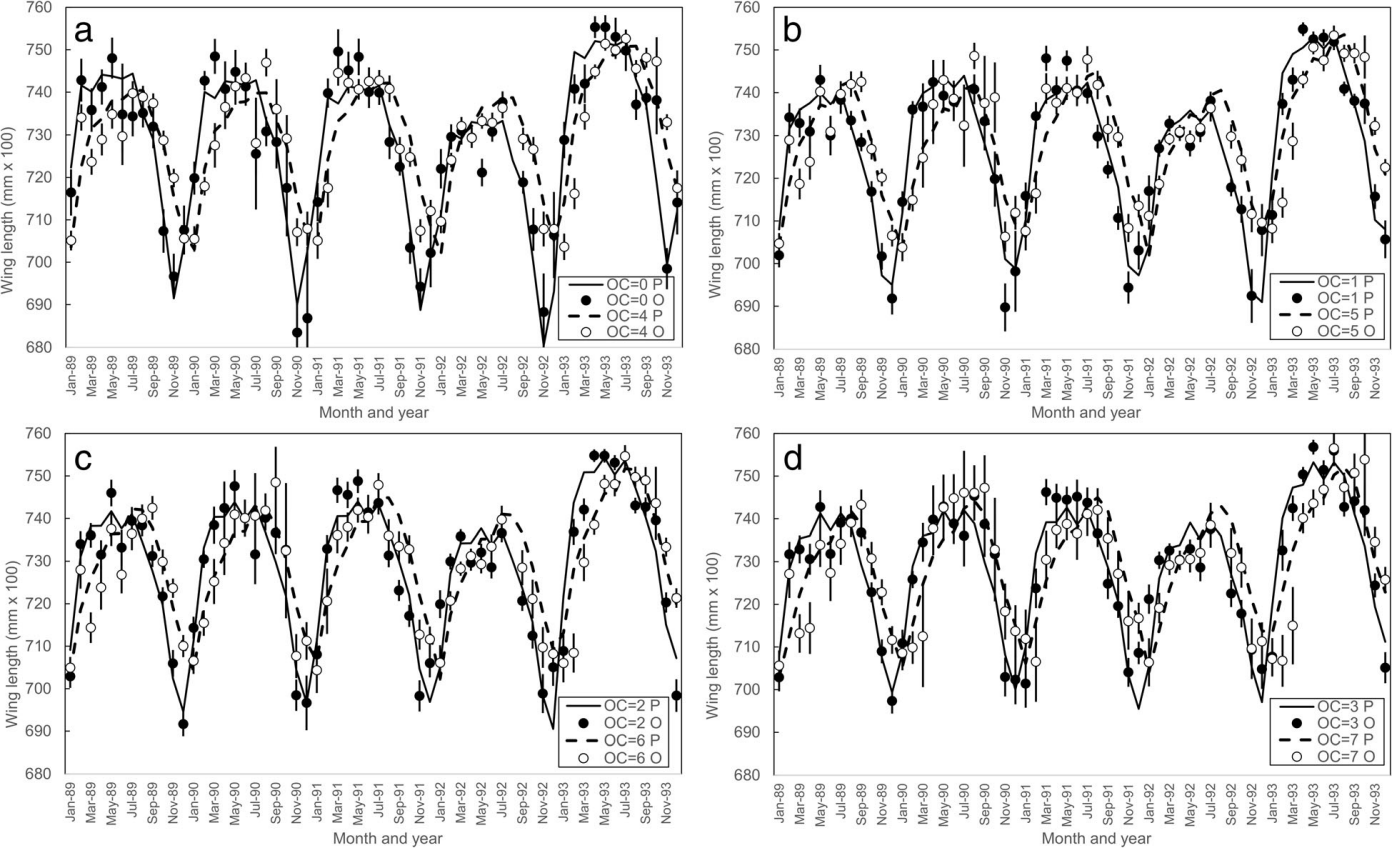
Mean wing lengths for flies in different ovarian age categories (OC) caught using odour-baited traps or the VET: **a** OC = 0 and 4; **b** OC = 1 and 5; **c** OC = 2 and 6; **d** OC = 3 and 7. Mean wing lengths for flies in different ovarian age categories (C) caught using odour-baited traps or the VET. Observed values (O) are the monthly means for all flies captured using the two sampling systems. Predicted values (P) are calculated using the results in Additional file 1: Table S1. Rekomitjie; January 1989 to December 1993

The results of the full multivariable analysis were used to predict the wing lengths for each ovarian category of flies captured in traps in each month across the 60 months of the study (Fig. 7). The sizes for flies caught using the VET were similarly calculated using the coefficients in the regression equation for method, and the interaction between capture month and method. Results for all ovarian categories were closely similar in showing that, in absolute terms, wing length hardly varied with capture method. For female *G. pallidipes* (Table 4), the average wing length of flies caught on the VET was just 0.0173 mm shorter than for flies caught in the trap, which is only 0.24% of the mean length of 7.078 mm. For *G. m. morsitans* the difference was 0.0297 mm, which is 0.48% of 6.183 mm (Table 4). These apparent variations due to capture method are much less than the total background variation. For example, with *G. pallidipes* the coefficient for the effect of month of capture varied between 32.77 in July and -1.53 in December, a difference of 34.30. This was 19.8 times greater that the bait effect of 1.73 (Table 4). For *G. m. morsitans* the shift between the same months was 27.85 - (-5.85) = 33.70, 11.3 times greater than the bait effect of 2.97.

## Discussion

Our results (Tables 2, 3; Fig. 3) confirm earlier indications that the availability of tsetse to stationary baits, as compared to mobile ones, is much greater for the taxa of larger wing size, i.e. greater for *G. pallidipes* than for *G. m. morsitans*, and greater for females than for males. The results provide little support, however, for the idea that larger individuals within a taxon are selectively attracted to stationary baits: subgroups of female *G. pallidipes* that were either larger or smaller than average showed no consistent size-related biases in such behaviour (Fig. 4). While multivariable analysis of the effects of month, year, fly age and capture method showed a statistically significant effect of bait type on fly size, the absolute magnitude of this effect was very small (Table 4) and much less than the total background variation. For example, with *G. pallidipes* the average seasonal effect (Table 4) was on average 20 times greater that the apparent bait effect, and for *G. m. morsitans* it was 11 times greater. Moreover, the mean size variation between the two species was 30–52 times the bait effect.

The present indications of wide background variations in fly size associated with season and year are consistent with published evidence [33–37]. There is also evidence that tsetse caught in localities just one or a few kilometres apart can differ in mean size and in the seasonal pattern of size variations [38, 39]. To this variability might be added the expectation that changes in host availability in isolated populations would affect the nutritional state of female tsetse and hence alter the size of offspring. Given the plethora of factors liable to affect the mean size of tsetse, we must thus join Glasgow & Bursell [36] in warning against accepting what might appear the simplest or most appealing interpretation of size differences between flies collected at different times and places, especially if the samples do not contain many tsetse, and are not obtained with systematic and comprehensive attention to what are likely to be the main sources of background variation.

Our results for *G. pallidipes* and *G. m. morsitans* thus provide no support for the idea that campaigns using (stationary) targets could cause any meaningful shift towards tsetse that are relatively small, immobile and unavailable to targets [11]. The ability to eradicate these species thus seems unimpeded by any size-bias in the availability of tsetse to these baits. This accords with the results of various successful target-based operations in Zimbabwe [40–42] and with the calculations of the probability of extinction resulting from such operations [43].

Targets have, however, often been used in areas where there was no intention or expectation of eliminating the flies, especially in those very common situations where invasion can occur from neighbouring untreated areas (e.g. [12, 41]), as would be the case, for example, if odour-baited targets were deployed in the neighbourhood of Rekomitjie Research Station. In such places, no control method of any sort can eliminate the flies unless invasion is specifically prevented. Targets can be particularly useful in providing an invasion barrier [17, 44], and consequently they are often used in association with other control techniques [45].

The situation might be different for *G. f. fuscipes*, which does not use odour in host location in the manner of *G. m. morsitans* and, particularly, *G. pallidipes*. The proportion of the population trapped per target is thus much smaller for *G. f. fuscipes*. The target density required to achieve a desired rate of population reduction is thus much higher for *G. f. fuscipes* than for the Zimbabwe species. This implies that *G. f. fuscipes* need not travel so far as *G. pallidipes* in order to find a target, so reducing any selection against large and relatively mobile individuals. Various considerations suggest, anyway, that more data need to be produced before one could unequivocally attribute the size changes recorded in the recent study on *G. f. fuscipes* in Kenya to the use of targets in the control campaign [11]. First, the possibility could not be excluded that increases in fly numbers following control operations in the study area were due to invasion from neighbouring areas [11]. If that were the case, it would be unclear whether there was a real change in the size of tsetse in the original population within the operational area, and how great that change might be, and what might have caused it. Secondly, the study involved comparing samples, captured using only stationary traps, collected over just three days in a two-month period with samples obtained during many days in a 19-month period, four to five years previously, with no sampling in-between.

Even if the results are accepted at face value, it appears that the 19-months of suppression due to targets deployed on Big Chamaunga Island reduced the mean size of female tsetse on the island by only about 1% [11]. This very small effect appears compatible with present evidence that, within a taxon, the mean sizes of tsetse available to mobile and stationary baits differ minimally compared to gross variations associated with sex, species, and environmental factors. It is also consistent with the suggestion that the size-associated responsiveness of tsetse is part of the innate behavioural repertoire of all flies of each taxon, which takes account of the average size of the tsetse within it [5]. Finally, it is consistent with Brady’s [8] demonstration that spontaneous activity is innate, and increases in intensity as the hunger cycle develops. The increased activity enhances the probability of locating stationary, as well as mobile, baits. Flies that systematically avoided behaviour that could lead to finding stationary baits would be more likely to starve, thereby reducing the likelihood of effective genetic selection in favour of such avoidance.

### Possible limitations of our work

Given that the flies used in our analysis were sampled up to 30 years ago, the question may arise as to the usefulness of our results in modern contexts. As indicated in the Methods section, Rekomitjie is situated within a very large protected area and there have been no major changes to the environment around Rekomitjie due to human settlement or agricultural activity. We cannot exclude the possibility of environmental changes due to climate and weather changes over the past few decades. Such changes would, however, only be important to our analysis if it affected the *relative* sizes of tsetse caught using mobile and stationary baits. Results from our study suggest that such effects are unlikely. Thus, as with most field situations in most environments, there are major, annual, cyclical changes in the weather at Rekomitjie, and these are associated with changes in vegetation and in animal abundance and distribution. During the course of the study there were also major differences between years in the temperatures measured at the station. As indicated by our results (Fig. 4), these changes are indeed associated with changes in the size of tsetse captured at different times of the year. The important point, however, is that the sizes of flies taken from our mobile and stationary sampling systems changed in very similar ways with time. There seems no reason to suggest, therefore, that any long-term environmental changes will have affected the conclusions of our study.

## Conclusions

Stationary odour-baited traps show a very small absolute bias in favour of larger flies within a taxon. There seems no reason to believe that stationary odour-baited targets are incapable of eliminating tsetse from suitably isolated situations, nor that they will affect materially the feeding behaviour of tsetse, nor that catches from stationary sampling devices will give seriously misleading indices of changes in tsetse population density.

## Additional file

Additional file 1: **Text S1.** Capture methods. **Figure S1.** Epsilon-trap as used for sampling tsetse species of the Morsitans group found in southern Africa. **Figure S2.** Vehicle-mounted electric target (VET). **Figure S3.** Artificial refuge - photograph and plan. **Text S2.** Tsetse life-cycle. **Text S3.** Ovarian dissection: estimation of fly age. **Figure S4.** Diagrammatic representation of the relative sizes of oocytes of female tsetse (*Glossina* spp) during successive ovulation cycles. Text S4 Climate profiles at Rekomitjie Research Station. **Figure S5.** Average monthly maximum and minimum temperatures and rainfall for the period 1 January 1989 to 31 December 1993 at Rekomitjie Research Station. **Table S1.** Multivariable analysis of the effects of ovarian age, month, year and method of capture, and interactions between age and the three other variables, on the mean wing length of female tsetse captured in the field at Rekomitjie Research Station, 1989–1993. (DOCX 961 kb)

## Abbreviations

CI: Confidence interval
CS: Centroid size
SACEMA: Department of Science and Technology/National Research Foundation Centre of Excellence in Epidemiological Modelling and Analysis
TRDW: Thoracic residual dry weight
UK: United Kingdom
UNESCO: United Nations Educational, Scientific and Cultural Organization
VET: Vehicle-mounted electric target
