# Supplementary material for: Wing length and host location in tsetse (Glossina spp.): implications for control using stationary baits
Source: Parasit Vectors. 2019 Jan 11;12:24. doi: 10.1186/s13071-018-3274-x (PMC6329045; doi:10.1186/s13071-018-3274-x)
Supplement: Supplementary file 1 — Text S1. Capture methods. Figure S1. Epsilon-trap as used for sampling tsetse species of the Morsitans group found in southern Africa. Figure S2. Vehicle-mounted electric target (VET). Figure S3. Artificial refuge - photograph and plan. Text S2. Tsetse life-cycle. Text S3. Ovarian dissection: estimation of fly age. Figure S4. Diagrammatic representation of the relative sizes of oocytes of female tsetse (Glossina spp) during successive ovulation cycles. Text S4 Climate profiles at Rekomitjie Research Station. Figure S5. Average monthly maximum and minimum temperatures and rainfall for the period 1 January 1989 to 31 December 1993 at Rekomitjie Research Station. Table S1. Multivariable analysis of the effects of ovarian age, month, year and method of capture, and interactions between age and the three other variables, on the mean wing length of female tsetse captured in the field at Rekomitjie Research Station, 1989–1993. (DOCX 961 kb) [file 13071_2018_3274_MOESM1_ESM.docx]

**Wing length and host location in tsetse (*Glossina* spp): implications for control using stationary baits**

John Hargrove, Sinead English, Stephen J. Torr, Jennifer Lord, Lee Rafuse Haines,

Cari van Schalkwyk, James Patterson and Glyn Vale

**Additional file 1: Text S1**

**Capture Methods**

i) Stationary mechanical traps, such as those described by Hargrove *et al*. (1995), baited with artificial host odour consisting of acetone (dispensed at 500 mg h^–1^), 1-octen-3-ol (0.4 mg h^–1^), 4-methyl phenol (0.8 mg h^–1^) and 3-n-propyl phenol (0.1 mg h^–1^) (Torr *et al.*, 1997). For convenience these devices as referred to in the main text simply as “traps”.

**Additional file 1: Figure S1**. **Epsilon-trap** As used for sampling Morsitans group species tsetse found in southern Africa.

ii) A vehicle-mounted electric target (VET) (Hargrove, 1999c), which consisted of an electrocuting grid, 1m tall and 2m long (Vale, 1974) mounted on the back of an open pick-up. The vehicle was driven at a speed of about 5 km/hr through tsetse habitat. Flies colliding with the net were killed, dropping to the floor of the pick-up where they were collected at 30 min intervals.

**Additional file 1:** **Figure S2**. **Vehicle-mounted electric target (VET)**

iii) Artificial refuges (Vale, 1971) were used for capturing tsetse that were analysed for levels of fat and haematin (Hargrove, 1999 a, b).

**
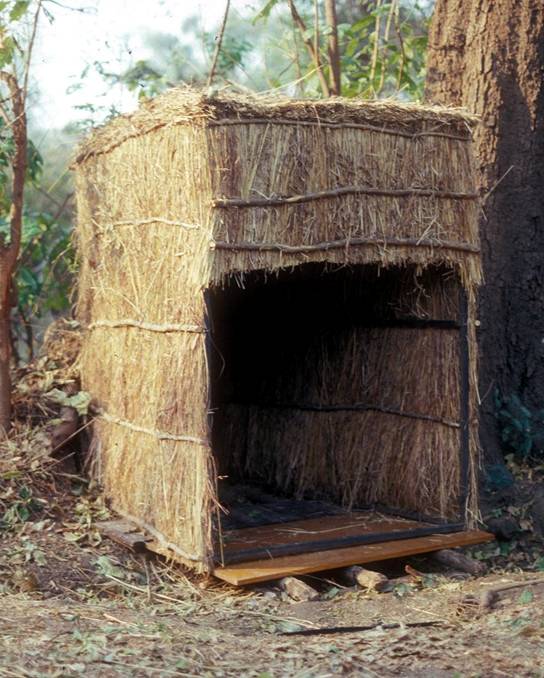
**

**
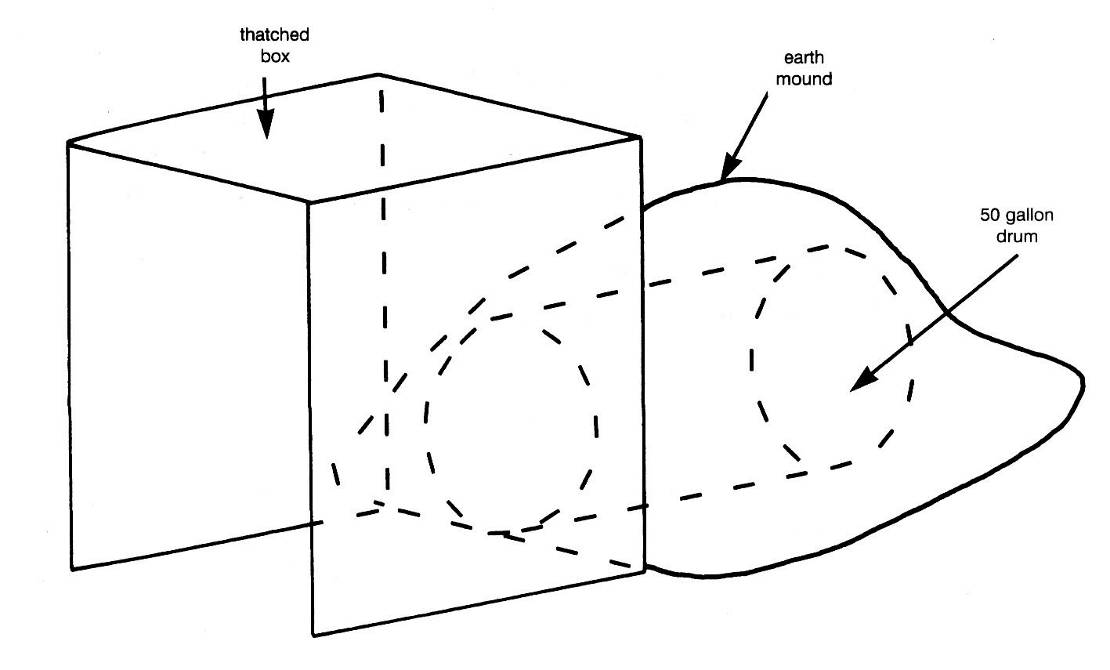
**

**Additional file 1:** **Figure S3**. **Artificial refuge – photograph and plan**

**Additional file 1:** **Text S2**

**Tsetse life cycle**

Unlike most Diptera, female tsetse flies (*Glossina* spp.) do not deposit eggs. Instead, in a process termed *adenotrophic viviparity*, a single fertilised egg is retained in the uterus during each pregnancy. After the egg hatches, three instars of the larval stage also develop in the uterus, fed by the mother via a modified uterine (“milk”) gland. A late third instar larva is eventually deposited, often weighing more than the female which has just deposited it. The larva is typically deposited on dry ground consisting of loose soil or sand, often mixed with vegetational litter. The larva burrows about 2.5 cm into this substrate and forms around itself, within minutes, a hard puparial case. The deposited larva does not, and does not need to, feed: the female provisions its offspring with all of the energy (in fat) and raw materials required to sustain the development right through the pupal stage to the point where an adult fly emerges from the puparial case.

The above reproductive process puts a huge physiological load on the adult female: she is able to keep this cost within reasonable bounds only by providing the larva she deposits with less energy and raw material than is required to produce a fully mature adult. Thus, while the teneral (*i.e.* unfed) adult that emerges from the puparial case has the full size of a mature adult, it has poorly developed flight musculature, and low fat reserves.

Both sexes of tsetse feel exclusively on blood, again setting them apart from almost all other free-flying arthropods. The first 2-3 blood-meals taken by young flies are used to redress the shortfalls in fat levels and flight muscle mass found in teneral flies. Thereafter the mature female uses blood to produce the “milk” required to nourish her own larvae.

All of the development periods referred to above are temperature dependent. The pupal phase increases from 20 days at 32^0^C to 100 days at 16^0^C (28 days at 25^0^C) (Phelps & Burrows, 1969), though few adults emerge at the extremes of temperature. Blood-meals are taken every 2 – 5 days, again depending on temperature, though there is no general agreement on the relationship between frequency and temperature (Hargrove, 1999a, b). There is also a weak relationship between temperature and the time between adult female emergence and first ovulation, which has been estimated to take a constant value of 8 days in the field at Rekomitjie (Hargrove, 1994). Thereafter the duration of pregnancy (the *inter-larval period*) increases from an estimated 7 days at 32^0^C to 12 days at 20^0^C (9 days at 25^0^C) (Hargrove, 1995).

**Additional file 1: Text S3**

**Ovarian dissection: estimation of fly age**

The ages of female tsetse flies can be estimated from the disposition and size of reproductive products in the ovaries and uterus. The method was first developed for mosquitoes by Detinova (1962), adapted the method for use on tsetse initially by Saunders (1960, 1962) and then improved by Challier (1965). The method uses the fact that ovarian and uterine structures show, as indicated from the life cycle (see above) easily identifiable, predictable and regularly timed changes.

Female tsetse have paired ovaries and each ovary has two ovarioles within which oocytes develop in strict order. Figure S4 illustrates the disposition of the four oocytes within the paired ovaries at different stages of the life of a female tsetse fly. In the normal fly, oocytes develop and are ovulated in the order: right inner (labelled A), left inner (C), right outer (B) and left outer (D). Examination of the ovaries through dissection, allows us to assign a female fly to an *ovarian category*, indicating the number of times that they had ovulated (Challier, 1965).

The oocytes of a fly that has ovulated four times have, however, the same relative sizes as a fly that has not yet ovulated (Figure S4). Categories 0 and 4 can, however, be distinguished because ovulation results in the ovariole bearing a follicular relic of the previous ovulation. A fly that has ovulated four times has a relic on each ovariole, whereas a fly in ovarian category zero has no relic on any ovariole. Similarly, flies in ovarian category 1 can be distinguished from flies in ovarian category 5; flies in ovarian category 2 can be distinguished from flies in ovarian category 6; and flies in ovarian category 3 can be distinguished from flies in ovarian category 7.

Unfortunately, only one relic is ever seen on an ovariole: further ovulations do not result in changes in the appearance of the relics and it is thus only possible for flies in ovarian categories 4, 5, 6 and 7 to estimate the number of ovulations modulo 4. For example, a fly in ovarian category 4 can have ovulated 4, 8, 12, 16 *. . .* , etc., times and similar statements apply to flies in ovarian categories 5, 6 and 7.

Estimates have been of the actual number of times flies in an ovarian category greater than 3 had ovulated, using information on the degree of fray of the wings of captured flies, which can be used as an independent estimate of fly age (Jackson, 1946). This technique has not been perfected, however, and this refinement is not attempted in the present work.

**

**

**Figure S4.** Diagrammatic representation of the relatives sizes of oocytes of female tsetse (*Glossina* spp) during successive ovulation cycles. Ovarioles ovulate in the strict order right inner (A), left inner (C), right outer (B) and left outer (D). It has been customary in the past to separate flies in ovarian category 0 (those that have not yet ovulated) into two groups (0a and 0b) depending on the size of the largest oocyte.

**Additional file 1: Text S4**

**Climate profiles at Rekomitjie Research Station**

Mean monthly maximum temperatures at Rekomitjie generally peaked in October (six times) or November (five times): an exception occurred in the hot season of 1991/1992 when the hottest month was in February 1992. Rekomitjie experiences a single rainy season with the vast majority of rain falling in the months of December – February. Temperatures then decline until July before the onset of the next hot dry season (Figure S5).

Local minima in catches often occurred in November and the declines in catch at these times are more likely to reflect real declines in the population due to increased mortality. In October and November activity is generally low during the heat of the day (Vale, 1971), but this means that feeding activity is concentrated during the last hours of the day (Hargrove & Brady, 1992), when the odour-baited traps were operated. The interpretation is complicated, however, by changes in the distribution of ovarian categories in samples of female tsetse as a function of season, capture method and tsetse species.

**Additional file 1:** **Figure S5.** Average monthly maximum and minimum temperatures and rainfall for the period 1 January 1989 to 31 December 1993 at Rekomitjie Research Station.

**Results**

**Additional file 1:** **Table S1.**  Multivariate analysis of the effects of ovarian age, month, year and method of capture, and interactions between age and the three other variables, on the mean wing length of female tsetse captured in the field at Rekomitjie Research Station, 1989 – 1993.

The lines immediately above the table provide the Stata code required for the analysis, which differs from the analyses used to produce the results shown in Table 4 of the main text only in the addition of the interaction terms highlighted in green.

***G. pallidipes***

**. regress wlm i.c i.cy i.cm i.md i.c##i.cm i.c##i.cy i.c##i.md if g==2 & s==2 & cy>88 & cy<94 & (mdp==4|md==30)**

Source | SS df MS Number of obs = 80,821

-------------+---------------------------------- F(135, 80685) = 252.55

Model | 16411217.8 135 121564.576 Prob > F = 0.0000

Residual | 38837060.3 80,685 481.341765 R-squared = 0.2970

-------------+---------------------------------- Adj R-squared = 0.2959

Total | 55248278.1 80,820 683.596611 Root MSE = 21.94

------------------------------------------------------------------------------

wlm | Coef. Std. Err. t P>|t| [95% Conf. Interval]

-------------+----------------------------------------------------------------

c |

1 | -14.32273 1.518969 -9.43 0.000 -17.2999 -11.34556

2 | -13.22893 1.501873 -8.81 0.000 -16.17259 -10.28527

3 | -14.5821 1.574001 -9.26 0.000 -17.66713 -11.49707

4 | -18.7122 1.464446 -12.78 0.000 -21.58251 -15.8419

5 | -18.3 1.484065 -12.33 0.000 -21.20876 -15.39124

6 | -18.67872 1.550532 -12.05 0.000 -21.71775 -15.63969

7 | -19.41282 1.709983 -11.35 0.000 -22.76437 -16.06126

|

cy |

90 | -1.536118 1.056945 -1.45 0.146 -3.607723 .5354865

91 | -2.796162 .9153638 -3.05 0.002 -4.590269 -1.002055

92 | -11.07657 .9134106 -12.13 0.000 -12.86685 -9.286288

93 | 7.874351 .9351681 8.42 0.000 6.041428 9.707274

|

cm |

February | 19.26918 1.341076 14.37 0.000 16.64068 21.89768

March | 17.80477 1.439617 12.37 0.000 14.98313 20.62641

April | 21.81557 1.325337 16.46 0.000 19.21792 24.41322

May | 21.41448 1.418958 15.09 0.000 18.63333 24.19563

June | 20.84172 1.353257 15.40 0.000 18.18935 23.4941

July | 22.04972 1.380795 15.97 0.000 19.34337 24.75607

August | 12.71914 1.422968 8.94 0.000 9.930134 15.50815

September | 6.592259 1.318004 5.00 0.000 4.008979 9.175538

October | -11.2687 1.766964 -6.38 0.000 -14.73193 -7.805461

November | -30.77377 1.716893 -17.92 0.000 -34.13887 -27.40867

December | -18.11601 1.89529 -9.56 0.000 -21.83076 -14.40125

|

md |

LR | -1.334009 .6333741 -2.11 0.035 -2.575418 -.0925999

|

c#cm |

1#February | 4.285582 1.675927 2.56 0.011 1.000776 7.570387

1#March | 10.19698 1.789694 5.70 0.000 6.689187 13.70476

1#April | 7.715017 1.65346 4.67 0.000 4.474246 10.95579

1#May | 10.47841 1.752438 5.98 0.000 7.043641 13.91317

1#June | 8.463093 1.704903 4.96 0.000 5.121495 11.80469

1#July | 10.02502 1.721409 5.82 0.000 6.651073 13.39897

1#August | 11.43014 1.760512 6.49 0.000 7.979546 14.88073

1#September | 9.618296 1.645007 5.85 0.000 6.394094 12.8425

1#October | 18.92696 2.098684 9.02 0.000 14.81355 23.04036

1#November | 19.96187 2.083959 9.58 0.000 15.87732 24.04641

1#December | 5.079614 2.292521 2.22 0.027 .5862882 9.572939

2#February | 2.680313 1.652786 1.62 0.105 -.5591358 5.919763

2#March | 11.31022 1.741433 6.49 0.000 7.897022 14.72342

2#April | 7.347712 1.632347 4.50 0.000 4.148323 10.5471

2#May | 11.18395 1.722907 6.49 0.000 7.807061 14.56083

2#June | 7.698883 1.69883 4.53 0.000 4.369188 11.02858

2#July | 9.890422 1.709159 5.79 0.000 6.540482 13.24036

2#August | 12.81419 1.757831 7.29 0.000 9.368848 16.25952

2#September | 12.02859 1.666248 7.22 0.000 8.762752 15.29442

2#October | 21.89246 2.077742 10.54 0.000 17.82011 25.96482

2#November | 24.07954 2.031518 11.85 0.000 20.09778 28.0613

2#December | 3.573465 2.184157 1.64 0.102 -.7074685 7.854399

3#February | 1.254452 1.727691 0.73 0.468 -2.13181 4.640714

3#March | 9.844653 1.813582 5.43 0.000 6.290044 13.39926

3#April | 6.49924 1.711355 3.80 0.000 3.144995 9.853486

3#May | 12.15467 1.805086 6.73 0.000 8.616713 15.69263

3#June | 8.735475 1.781038 4.90 0.000 5.244652 12.2263

3#July | 11.49825 1.81514 6.33 0.000 7.940591 15.05592

3#August | 17.77753 1.879527 9.46 0.000 14.09367 21.46139

3#September | 14.86202 1.785165 8.33 0.000 11.36311 18.36093

3#October | 25.25612 2.150312 11.75 0.000 21.04153 29.47072

3#November | 30.51445 2.090037 14.60 0.000 26.41799 34.61091

3#December | 9.68516 2.217514 4.37 0.000 5.338847 14.03147

4#February | -.3502135 1.625851 -0.22 0.829 -3.536871 2.836444

4#March | 10.14601 1.696396 5.98 0.000 6.821083 13.47093

4#April | 8.424229 1.590468 5.30 0.000 5.306922 11.54154

4#May | 13.02504 1.683172 7.74 0.000 9.726029 16.32404

4#June | 13.88012 1.649815 8.41 0.000 10.64649 17.11374

4#July | 14.65785 1.6843 8.70 0.000 11.35663 17.95906

4#August | 24.06196 1.753887 13.72 0.000 20.62435 27.49957

4#September | 23.71881 1.632156 14.53 0.000 20.5198 26.91782

4#October | 35.56407 2.034721 17.48 0.000 31.57603 39.55211

4#November | 42.54837 1.954549 21.77 0.000 38.71746 46.37927

4#December | 21.34566 2.088361 10.22 0.000 17.25248 25.43883

5#February | -4.46364 1.675042 -2.66 0.008 -7.746712 -1.180569

5#March | 6.238427 1.7478 3.57 0.000 2.81275 9.664104

5#April | 6.288094 1.631618 3.85 0.000 3.090133 9.486056

5#May | 11.71904 1.708997 6.86 0.000 8.369418 15.06866

5#June | 11.51569 1.680858 6.85 0.000 8.221223 14.81016

5#July | 15.50403 1.735152 8.94 0.000 12.10314 18.90491

5#August | 26.00693 1.800728 14.44 0.000 22.47751 29.53634

5#September | 25.98667 1.685042 15.42 0.000 22.684 29.28934

5#October | 35.73321 2.082813 17.16 0.000 31.65091 39.81551

5#November | 42.73317 1.981989 21.56 0.000 38.84849 46.61786

5#December | 24.18732 2.10814 11.47 0.000 20.05538 28.31926

6#February | -4.335235 1.777888 -2.44 0.015 -7.819883 -.8505859

6#March | 4.487328 1.825543 2.46 0.014 .9092762 8.06538

6#April | 5.504406 1.722725 3.20 0.001 2.127877 8.880935

6#May | 11.3632 1.797368 6.32 0.000 7.840371 14.88603

6#June | 11.98495 1.781964 6.73 0.000 8.492317 15.47759

6#July | 16.55087 1.84546 8.97 0.000 12.93378 20.16796

6#August | 25.6609 1.917563 13.38 0.000 21.90248 29.41931

6#September | 27.58996 1.817248 15.18 0.000 24.02817 31.15176

6#October | 37.55697 2.203081 17.05 0.000 33.23895 41.875

6#November | 46.23633 2.069898 22.34 0.000 42.17934 50.29331

6#December | 24.88213 2.172462 11.45 0.000 20.62412 29.14014

7#February | -9.503891 2.037296 -4.66 0.000 -13.49698 -5.510804

7#March | 1.568938 2.071915 0.76 0.449 -2.492003 5.629878

7#April | 4.23233 1.944998 2.18 0.030 .4201469 8.044512

7#May | 8.547906 2.025332 4.22 0.000 4.578268 12.51754

7#June | 10.82743 2.061929 5.25 0.000 6.786064 14.8688

7#July | 14.98299 2.11774 7.07 0.000 10.83223 19.13374

7#August | 26.10021 2.17706 11.99 0.000 21.83318 30.36723

7#September | 29.12344 2.08439 13.97 0.000 25.03805 33.20883

7#October | 37.81934 2.446025 15.46 0.000 33.02515 42.61353

7#November | 47.71022 2.2892 20.84 0.000 43.2234 52.19703

7#December | 27.7697 2.337646 11.88 0.000 23.18793 32.35147

c#cy |

1 90 | 5.389625 1.322695 4.07 0.000 2.797152 7.982099

1 91 | 5.074844 1.126934 4.50 0.000 2.866061 7.283627

1 92 | 7.062931 1.120682 6.30 0.000 4.866402 9.259461

1 93 | 5.146367 1.118201 4.60 0.000 2.9547 7.338034

2 90 | 3.654832 1.300346 2.81 0.005 1.106163 6.203501

2 91 | 5.102403 1.116854 4.57 0.000 2.913377 7.29143

2 92 | 7.050949 1.099242 6.41 0.000 4.896443 9.205455

2 93 | 4.757422 1.110218 4.29 0.000 2.581403 6.933441

3 90 | 2.204017 1.342223 1.64 0.101 -.4267312 4.834766

3 91 | 5.090993 1.155984 4.40 0.000 2.825272 7.356714

3 92 | 8.844696 1.135902 7.79 0.000 6.618335 11.07106

3 93 | 4.026325 1.148097 3.51 0.000 1.776062 6.276587

4 90 | .9887353 1.275409 0.78 0.438 -1.511058 3.488528

4 91 | 4.549313 1.090844 4.17 0.000 2.411266 6.687361

4 92 | 9.38952 1.06881 8.79 0.000 7.29466 11.48438

4 93 | 2.585388 1.090947 2.37 0.018 .4471398 4.723637

5 90 | .6879222 1.31262 0.52 0.600 -1.884804 3.260649

5 91 | 5.010765 1.118475 4.48 0.000 2.818562 7.202969

5 92 | 8.862094 1.090084 8.13 0.000 6.725536 10.99865

5 93 | 2.971287 1.110021 2.68 0.007 .7956529 5.14692

6 90 | 2.018703 1.372919 1.47 0.141 -.6722099 4.709616

6 91 | 5.614826 1.189505 4.72 0.000 3.283405 7.946247

6 92 | 9.826723 1.143135 8.60 0.000 7.586185 12.06726

6 93 | 1.579298 1.166234 1.35 0.176 -.7065129 3.865109

7 90 | 3.98991 1.573846 2.54 0.011 .9051825 7.074638

7 91 | 6.02689 1.363429 4.42 0.000 3.354579 8.699202

7 92 | 12.31091 1.293151 9.52 0.000 9.77634 14.84548

7 93 | 2.438609 1.319285 1.85 0.065 -.1471803 5.024399

|

c#md |

1#LR | -1.980379 .8809569 -2.25 0.025 -3.707048 -.253709

2#LR | -.3637051 .9010981 -0.40 0.686 -2.129851 1.402441

3#LR | .0103385 .9731702 0.01 0.992 -1.897069 1.917746

4#LR | -1.428768 .8817611 -1.62 0.105 -3.157014 .2994783

5#LR | -1.734304 .9378152 -1.85 0.064 -3.572416 .1038073

6#LR | -2.38084 1.038233 -2.29 0.022 -4.415769 -.3459105

7#LR | -2.386928 1.295637 -1.84 0.065 -4.926368 .1525113

|

_cons | 722.3307 1.253603 576.20 0.000 719.8736 724.7877

------------------------------------------------------------------------------

***G. m. morsitans***

. regress wlm i.c i.cy i.cm i.md **i.c##i.cm** if g==1 & s==2 & cy>88 & cy<94 & (mdp==4|md==30)

Source | SS df MS Number of obs = 7,806

-------------+---------------------------------- F(100, 7705) = 29.97

Model | 1334607.72 100 13346.0772 Prob > F = 0.0000

Residual | 3430851.83 7,705 445.276032 R-squared = 0.2801

-------------+---------------------------------- Adj R-squared = 0.2707

Total | 4765459.55 7,805 610.564965 Root MSE = 21.102

------------------------------------------------------------------------------

wlm | Coef. Std. Err. t P>|t| [95% Conf. Interval]

-------------+----------------------------------------------------------------

c |

1 | -8.322033 3.140385 -2.65 0.008 -14.47804 -2.166024

2 | -8.589805 3.096603 -2.77 0.006 -14.65999 -2.519622

3 | -10.74832 3.446719 -3.12 0.002 -17.50483 -3.991815

4 | -14.34612 3.000567 -4.78 0.000 -20.22805 -8.464195

5 | -20.31632 3.155673 -6.44 0.000 -26.5023 -14.13034

6 | -15.07461 3.483734 -4.33 0.000 -21.90367 -8.24554

7 | -23.65974 5.099388 -4.64 0.000 -33.65592 -13.66355

|

cy |

90 | 2.459155 1.099289 2.24 0.025 .3042497 4.61406

91 | 5.25902 .8361948 6.29 0.000 3.61985 6.898189

92 | -2.124311 .9467989 -2.24 0.025 -3.980295 -.268328

93 | 9.154773 .9505918 9.63 0.000 7.291355 11.01819

|

cm |

February | 6.955612 3.216089 2.16 0.031 .6512039 13.26002

March | 19.00345 3.801358 5.00 0.000 11.55176 26.45515

April | 27.58812 4.137147 6.67 0.000 19.47819 35.69806

May | 8.125123 5.016354 1.62 0.105 -1.708296 17.95854

June | 11.92369 3.268998 3.65 0.000 5.515571 18.33182

July | 18.58378 2.922265 6.36 0.000 12.85534 24.31221

August | 10.25987 3.39513 3.02 0.003 3.604491 16.91525

September | 6.516633 2.999607 2.17 0.030 .6365878 12.39668

October | -8.087055 3.625371 -2.23 0.026 -15.19377 -.9803407

November | -26.96295 3.709576 -7.27 0.000 -34.23472 -19.69117

December | -25.74806 4.303838 -5.98 0.000 -34.18476 -17.31137

|

md |

LR | -3.304438 .6436793 -5.13 0.000 -4.566225 -2.042652

|

c#cm |

1#February | 9.374409 4.291452 2.18 0.029 .9619968 17.78682

1#March | 1.596805 4.985293 0.32 0.749 -8.175724 11.36933

1#April | -8.193848 5.312532 -1.54 0.123 -18.60785 2.220159

1#May | 10.45835 6.078187 1.72 0.085 -1.45655 22.37325

1#June | 7.251285 4.287912 1.69 0.091 -1.154189 15.65676

1#July | 9.210576 4.000291 2.30 0.021 1.368918 17.05223

1#August | 7.312655 4.533524 1.61 0.107 -1.574286 16.1996

1#September | 7.134864 4.13876 1.72 0.085 -.9782308 15.24796

1#October | 10.28588 4.73474 2.17 0.030 1.0045 19.56726

1#November | 12.16286 4.885129 2.49 0.013 2.586676 21.73904

1#December | 9.977852 5.34298 1.87 0.062 -.4958413 20.45154

2#February | 3.964859 4.40235 0.90 0.368 -4.664943 12.59466

2#March | -3.068328 5.187815 -0.59 0.554 -13.23786 7.101199

2#April | -4.799034 5.425436 -0.88 0.376 -15.43436 5.836295

2#May | 14.13916 6.526412 2.17 0.030 1.345614 26.9327

2#June | 9.590974 4.407816 2.18 0.030 .9504554 18.23149

2#July | 6.34897 3.996565 1.59 0.112 -1.485385 14.18332

2#August | 8.733917 4.567936 1.91 0.056 -.2204797 17.68831

2#September | 8.794998 4.110765 2.14 0.032 .7367815 16.85321

2#October | 15.18465 4.646718 3.27 0.001 6.075824 24.29348

2#November | 14.08226 4.863485 2.90 0.004 4.548509 23.61602

2#December | 11.55047 5.182407 2.23 0.026 1.391545 21.7094

3#February | 1.05923 4.846586 0.22 0.827 -8.441397 10.55986

3#March | -3.485722 5.51203 -0.63 0.527 -14.2908 7.319356

3#April | -2.123461 6.222541 -0.34 0.733 -14.32133 10.07441

3#May | 11.97311 7.063544 1.70 0.090 -1.873355 25.81958

3#June | 13.61687 4.698444 2.90 0.004 4.406637 22.82709

3#July | 10.5988 4.320526 2.45 0.014 2.129391 19.0682

3#August | 11.99977 4.913579 2.44 0.015 2.367822 21.63172

3#September | 15.3366 4.402661 3.48 0.000 6.706192 23.96702

3#October | 18.91421 4.991217 3.79 0.000 9.130062 28.69835

3#November | 18.62635 5.28019 3.53 0.000 8.275741 28.97696

3#December | 15.58782 5.408944 2.88 0.004 4.984815 26.19082

4#February | -1.019057 4.482431 -0.23 0.820 -9.805842 7.767727

4#March | 4.077941 5.0949 0.80 0.424 -5.909449 14.06533

4#April | -2.36674 5.522897 -0.43 0.668 -13.19312 8.45964

4#May | 22.35981 6.244014 3.58 0.000 10.11985 34.59977

4#June | 14.70672 4.181179 3.52 0.000 6.510473 22.90297

4#July | 12.53271 3.878837 3.23 0.001 4.929139 20.13629

4#August | 20.16405 4.381867 4.60 0.000 11.5744 28.7537

4#September | 22.15368 3.958735 5.60 0.000 14.39348 29.91387

4#October | 29.97692 4.563106 6.57 0.000 21.032 38.92185

4#November | 35.28084 4.719416 7.48 0.000 26.0295 44.53218

4#December | 26.40144 4.980776 5.30 0.000 16.63776 36.16511

5#February | -1.329518 4.562111 -0.29 0.771 -10.2725 7.61346

5#March | -.319553 5.240675 -0.06 0.951 -10.5927 9.953595

5#April | 5.111792 5.890671 0.87 0.386 -6.435524 16.65911

5#May | 28.10835 6.679569 4.21 0.000 15.01458 41.20213

5#June | 19.59826 4.426451 4.43 0.000 10.92121 28.27531

5#July | 15.77862 4.070756 3.88 0.000 7.798829 23.75841

5#August | 21.7873 4.568196 4.77 0.000 12.83239 30.74221

5#September | 27.87405 4.172313 6.68 0.000 19.69519 36.05292

5#October | 34.97511 4.760038 7.35 0.000 25.64415 44.30608

5#November | 34.24756 4.906634 6.98 0.000 24.62923 43.8659

5#December | 34.70191 5.138293 6.75 0.000 24.62946 44.77436

6#February | -8.079591 5.016825 -1.61 0.107 -17.91393 1.754751

6#March | -6.61949 5.90206 -1.12 0.262 -18.18913 4.950153

6#April | -2.79301 7.095114 -0.39 0.694 -16.70136 11.11534

6#May | 18.29281 6.598629 2.77 0.006 5.3577 31.22792

6#June | 16.4798 4.843654 3.40 0.001 6.984921 25.97468

6#July | 9.583898 4.428643 2.16 0.030 .9025543 18.26524

6#August | 21.79337 5.148962 4.23 0.000 11.7 31.88674

6#September | 19.70907 4.636941 4.25 0.000 10.6194 28.79874

6#October | 32.22188 5.296555 6.08 0.000 21.83919 42.60457

6#November | 39.69076 5.304013 7.48 0.000 29.29345 50.08807

6#December | 27.42127 5.541162 4.95 0.000 16.55908 38.28345

7#February | 7.956546 7.195898 1.11 0.269 -6.149371 22.06246

7#March | -4.090445 7.516279 -0.54 0.586 -18.8244 10.64351

7#April | 7.623421 9.679704 0.79 0.431 -11.35143 26.59827

7#May | 23.77842 9.538943 2.49 0.013 5.079499 42.47734

7#June | 21.71282 6.794387 3.20 0.001 8.393974 35.03166

7#July | 15.43391 6.533478 2.36 0.018 2.626516 28.2413

7#August | 39.39632 7.57604 5.20 0.000 24.54522 54.24742

7#September | 35.1279 6.708942 5.24 0.000 21.97655 48.27925

7#October | 45.89276 7.384091 6.22 0.000 31.41793 60.36758

7#November | 50.42339 7.423761 6.79 0.000 35.8708 64.97598

7#December | 35.4303 7.27964 4.87 0.000 21.16023 49.70038

|

_cons | 628.5163 2.357061 266.65 0.000 623.8958 633.1368

------------------------------------------------------------------------------

**References**

**Challier, A.** (1965) Amélioration de la méthode de détermination de l'âge physiologique des glossines. Études faites sur *Glossina palpalis palpalis* Vanderplank, 1949. *Bulletin de la Société de Pathologie Exotique*, 58, 250-259.

**Detinova, T. S.** (1962) Age-grouping methods in Diptera of medical importance with special reference to some vectors of malaria. *Monograph Series of the World Health Organisation*. **47**,13-191.

**Hargrove, J. W.** (1994) Reproductive rates of tsetse flies in the field in Zimbabwe. *Physiological Entomology* **19**, 307-318.

**Hargrove, J.W.** (1995) Towards a general rule for estimating the day of pregnancy of field-caught tsetse flies. *Physiological Entomology* **20**, 213-223.

**Hargrove, J. W.** (1999a) Nutritional levels of female tsetse *Glossina pallidipes* from artificial refuges. *Medical and Veterinary Entomology*, **13**, 150-164.

**Hargrove, J. W.** (1999b) Lifetime changes in the nutritional characteristics of female tsetse flies *Glossina pallidipes* caught in odour-baited traps. *Medical and Veterinary Entomology*, **13**, 165-176.

**Hargrove, J. W.** (1999c) Reproductive abnormalities in field tsetse flies in Zimbabwe. *Entomologia Experimentalis et Applicata*, 92, 89-99.

**Hargrove, J. W. & Brady, J.** (1992) Activity rhythms of tsetse flies (*Glossina* spp.) (Diptera: Glossinidae) at low and high temperatures in nature. *Bulletin of Entomological Research* **82**, 321-326.

**Hargrove, J. W., Holloway, M.T.P., Vale, G. A., Gough, A.J.E. & Hall, D.J.** (1995). Catches of tsetse flies (*Glossina* spp.) (Diptera: Glossinidae) from traps baited with large doses of natural and synthetic host odour. *Bulletin of Entomological Research*, **85**, 215-227.

**Jackson, C. H. N.** (1946) An artificially isolated generation of tsetse flies (Diptera). *Bulletin of Entomological Research*, **37**, 291-299.

**Phelps, R.J. & Burrows, P. M.** (1969a) Puparial duration in *Glossina morsitans orientalis* under conditions of constant temperature. *Entomologia Experimentalis et Applicata*, **12**, 33-43.

**Saunders, D.S.** (1960) The ovulation cycle in *Glossina morsitans* Westwood (Diptera: Muscidae) and a possible method of age determination for female tsetse flies by the examination of their ovaries. *Transaction of the Royal Entomological Society London*, **112**, 221-238.

**Saunders, D.S.** (1962) Age determination for female tsetse flies and the age compositions of samples of *Glossina pallidipes* Aust., *G. palpalis fuscipes* Newst. and *G. brevipalpis* Newst. *Bulletin of Entomological Research*, **53**, 579-595.

**Torr, S. J. Hall, D., R., Phelps, R. J., & Vale, G. A.** (1997) Methods for dispensing odour attractants for tsetse flies (Diptera: Glossinidae). *Bulletin of Entomological Research* **87**, 299-311.

**Vale, G.A.** (1971) Artificial refuges for tsetse flies (*Glossina* spp.). *Bulletin of Entomological Research*, 61, 331-350.

**Vale, G.A.** (1974) The responses of tsetse flies (Diptera: Glossinidae) to mobile and stationary baits. *Bulletin of Entomological Research*, 64, 545-588.
